# Editorial: Molecular mechanisms underlying polycystic kidney disease: from the smallest bricks to the big scenario

**DOI:** 10.3389/fmolb.2024.1429206

**Published:** 2024-05-21

**Authors:** Annarita Di Mise, Michael J. Caplan, Giovanna Valenti

**Affiliations:** ^1^ Department of Biosciences, Biotechnologies and Environment, University of Bari Aldo Moro, Bari, Italy; ^2^ Department of Cellular and Molecular Physiology, Yale University School of Medicine, New Haven, CT, United States

**Keywords:** autosomal dominant polycystic kidney disease (ADKPD), autosomal recessive polycystic kidney disease (ARPKD), polycystins, primary cilium, CKD, chronic kidney disease

Polycystic Kidney Disease (PKD) is a genetic disorder characterized by the development and progressive enlargement of fluid-filled cysts in the kidney. It is caused by mutations in one of two genes, *PKD1*, encoding polycystin-1 (PC-1), or *PKD2*, encoding polycystin-2 (PC-2), causing Autosomal Dominant Polycystic Kidney Disease (ADPKD), or mutations in the *PKHD1* gene, encoding fibrocystin, leading to Autosomal Recessive Polycystic Kidney Disease (ARPKD) (Hughes et al., 1995; Mochizuki et al., 1996; Bergmann et al., 2004). Cyst growth alters renal structure and leads to kidney enlargement, causing impaired function and potentially lethal organ failure.

Due to its complex genetic background and its outcome on renal function and other organs, efforts to understand PKD require a multifaceted approach. The present Research Topic contains 9 noteworthy articles describing recent progress and emerging insights from PKD research, shedding light on novel therapeutic approaches and promising targets for intervention.

ADPKD prevalence is reported to be between 1 in 400 and 1 in 1,000 births, resulting in kidney failure in 50% of patients by 60 years of age (Bergmann et al., 2018). ARPKD instead is a much rarer disease characterized by a perinatal, pediatric onset with kidney impairment described in 60% of patients by 20 years of age (Bergmann et al., 2018). Most ARPKD children are hypertensive in the first year of life. Conversely, a significantly lower percentage of ADPKD patients develop hypertension during childhood, but this percentage is probably underestimated. In the review by Lucchetti et al., the authors analyze the available pediatric studies and experience-based observations on cardiovascular impairment associated with both PKD forms. Since early onset hypertension (before the age of 35 years) represents a risk factor for fast progression of chronic kidney disease (CKD), the authors underline that an early hypertension treatment may slow down the progression of the disease and reduce cardiovascular complications.

PKD is listed among ciliopathies—disorders affecting primary cilia proteins (Ta et al., 2020). Polycystins (PCs) localize to the primary cilium in the kidney tubular epithelial cells and loss of PCs function results in loss of lumen diameter control leading to luminal expansion and cyst formation (Luo et al., 2023). Specifically, polycystin function is believed to embody an inhibitory activity that suppresses the cilia-dependent cyst activation (CDCA) signal (Luo et al., 2023). In this respect, in the review by Walker et al., the authors discuss the current model of the CDCA mechanism in ADPKD and consider the possible roles of ciliary and extraciliary polycystins in regulating CDCA. Moreover, they hypothesize the existence of cilia-localized components of CDCA (cCDCA) and cilia-localized cyst inhibition (CLCI) signals, proposing TULP3 cargoes as potential cilia-localized components that determine cystogenesis in kidneys during development and in adult mice.

Recent studies have highlighted that PC1’s capacity to modulate G protein signaling may play a crucial role in preventing the development of renal cysts, although the exact mechanisms are still being investigated (Fedeles et al., 2014; Wu et al., 2016; Parnell et al., 2018). PC1 may be involved in the control of GPCR-mediated signaling pathways based on the structural and functional similarities between polycystin-1 and the family of cell adhesion GPCRs, such as the presence of a conserved GPCR proteolysis site (GPS) (Maser and Calvet, 2020). The evidence for PC1 GPCR-like activity, the role of GPS cleavage in controlling PC1 GPCR function, and the possible interaction between PC1 GPCR-like activity and the regulation of polycystin complex channel properties have been reviewed by Maser et al.

PKD progression can be exacerbated by the presence of renal innate immune cells (Zimmerman et al., 2020). This interesting aspect is examined in the review by Agborbesong et al., focusing on epigenetic regulation, inflammation, and cell death as molecular mechanisms underlying ADPKD. It draws attention to the intricate interplay that drives cyst formation and disease progression, involving *PKD* gene alterations, epigenetic changes, inflammatory responses, and different forms of cell death. The inflammasome system responds to stimuli such as cellular damage by activating Caspase-1, and producing essential mediators of the inflammatory pathway, including IL-1β and IL-18. In the original research article by Swenson-Fields et al., the authors demonstrate that Caspase-1 knockout markedly reduced the onset of PKD in female mice, indicating sex-specific immunological responses, showing for the first time that the activated Caspase-1/inflammasome promotes cyst expansion and disease progression in PKD, particularly in females.

Currently, there is no cure for PKD other than renal transplantation (Dennis et al., 2023). Tolvaptan is the only drug approved by FDA proven to slow eGFR decline in ADPKD patients at the risk of rapid disease progression. Widespread use of tolvaptan is limited by the substantial aquaretic effects that it produces and by the potential for liver toxicity (Zhou and Torres, 2023). Recent advances in understanding the pathophysiology of PKD have led to new approaches to treatment via targeting different signaling pathways (Zhou and Torres, 2023).

The original research article by Hallows et al. investigates the potential therapeutic effects of bempedoic acid (BA), an ATP citrate-lyase (ACLY) inhibitor. The authors demonstrate that BA inhibited cyst growth and improved mitochondrial function *in vitro*, and reduced disease severity *in vivo*, suggesting BA as a promising therapy for PKD, having beneficial effects alone and associated with tolvaptan.

The review article by Zhou and Torres explores the emerging therapies for ADPKD with a focus on cAMP signaling. It discusses the role of cAMP and PKA signaling in ADPKD pathogenesis and the potential of targeting downstream pathways beyond cAMP production for therapeutic interventions. Over the past years, several *in vitro* and animal studies have shown that metabolic reprogramming might be a general feature of PKD (Hopp et al., 2022). Glucose metabolism is defective in ADPKD, with cystic cells reprogrammed to favor aerobic glycolysis. In addition to glucose, altered amino acid metabolism, reduced fatty acid oxidation, and dysregulated lipid metabolism have also been identified as key features of PKD (Hopp et al., 2022). In the mini-review by Bacaj and Pokai, the authors discuss metabolism-based approaches for ADPKD treatment, highlighting the role of metabolic reprogramming in cyst growth, specifically upregulated mTOR and c-Myc pathways, and the potential for targeting these pathways as therapeutic approaches.

Obesity and overweight are very common in ADPKD patients and represent independent risk factors for the disease advancement. In this regard, Iliuta et al. examine the shared pathobiology between ADPKD and obesity, emphasizing the role of reduced AMPK activity and increased mTOR signaling. The pharmacological activation of AMPK is discussed as a promising approach to treat both ADPKD and obesity-related kidney disease.

In conclusion, the present Research Topic provides an overview of the ongoing efforts to unravel the complex interaction of molecular signaling pathways associated with PKD progression, exploring innovative therapeutic approaches to improve patient outcomes.
